# Association of HLA Haplotypes with Autoimmune Pathogenesis in Newly Diagnosed Type 1 Romanian Diabetic Children: A Pilot, Single-Center Cross-Sectional Study

**DOI:** 10.3390/life14060781

**Published:** 2024-06-20

**Authors:** Amalia Ioana Arhire, Sorin Ioacara, Teodora Papuc, Miruna Sânziana Chiper, Irina Monica Dutescu, Ana Moise, Ioana Roxana Badea, Suzana Florea, Adelina Vlad, Simona Fica

**Affiliations:** 1 General Medicine Faculty, “Carol Davila” University of Medicine and Pharmacy, 050474 Bucharest, Romania; amalia.arhire@drd.umfcd.ro (A.I.A.); adelina.vlad@umfcd.ro (A.V.); simona.fica@umfcd.ro (S.F.); 2 Department of Pediatric Endocrinology and Diabetes, Elias Emergency University Hospital, 011461 Bucharest, Romania; papuc.teodora@gmail.com (T.P.); miruna.chiper@gmail.com (M.S.C.); 3 HLA Laboratory, “C.T. Nicolau” National Institute of Blood Transfusion, 011154 Bucharest, Romania; lab.hla-inht@donare-sange.ro (I.M.D.); moiseaana@yahoo.com (A.M.); ioanabroxana@yahoo.com (I.R.B.); 4 Immunology Laboratory, Elias Emergency University Hospital, 011461 Bucharest, Romania; suzana.sv@gmail.com

**Keywords:** type 1 diabetes, HLA, autoimmune, vitamin D, Hashimoto thyroiditis, celiac disease

## Abstract

Background: The increasing incidence of autoimmune diseases in type 1 diabetes mellitus (T1DM) patients highlights the influence of human leukocyte antigen (HLA) haplotypes on their development. This study aims to determine genetic predisposition to autoimmune diseases in T1DM patients, including thyroid disease and celiac diseases, and explore its correlation with vitamin D deficiency. Methods: A cross-sectional study involving thirty-six T1DM children was conducted. Typing was performed for the HLA A, B, C, DP, DR, and DQ loci. Regression analysis linked DR-DQ haplotypes to T1DM and the associated conditions. Results: The most frequent predisposing alleles and haplotypes were HLA-DR3 (70.27%), DQ2 (70.27%), DR3-DQ2 (70.27%), DQB1*02:01 (70.27%), A02 (54.05%), whereas the most prevalent protecting allele was DPB1*04:01 (52.63%). Positive correlations were observed between positive anti-thyroid peroxidase antibodies and the absence of protective alleles (DPB1*04:02, *p* = 0.036; DPB1*04:01, *p* = 0.002). Associations were found between the absence of DPB1*04:01 and anti-thyroglobulin antibodies (*p* = 0.03). HLA allele DPB1*03:01 was linked with vitamin D deficiency (*p* = 0.021). Positive anti-transglutaminase antibodies correlated with C03:03 (*p* = 0.026) and DRB1*04:01-DQA1*03-DQB1*03:01 (*p* < 0.0001) and the lack of DQA1*01:03-DQB1*06:03-DRB1*13:01 (*p* < 0.0001). Conclusions: The predisposing T1DM haplotypes were associated with the presence of anti-transglutaminase and anti-thyroid antibodies, indicating a genetic predisposition to autoimmune diseases.

## 1. Introduction

Type 1 diabetes mellitus (T1DM) is a complex condition with a deeply genetic etiology, necessitating personalized management to achieve optimal therapeutic outcomes [1]. Studies indicate a steady increase in the incidence rate of T1DM in Romania, from 4.7 per 100,000 person-years in 1996 to 11.0 in 2015, with an annual growth rate of 5.1%. This alarming trend suggests that the prevalence of T1DM could double every 14 years if current rates continue [2].

Globally, T1DM represents an escalating burden, significantly impacting youth, underscoring the urgent need for a better understanding of its genetic underpinnings. At the core of this genetic landscape is the major histocompatibility complex (MHC), accounting for approximately 40–50% of the familial aggregation of T1DM, encoding gene products initially identified on the surfaces of white blood cells as leukocyte antigens, known as the human leukocyte antigen (HLA) complex [1,3,4]. Class I alleles in T1DM susceptibility have shown significant associations with HLA-A, -B, and -C loci. The most prominent predisposing variant is B39:06, followed by other noteworthy T1DM-associated alleles including A24:02, A02:01, B18:01, and C05:01 [5].

HLA allele and haplotype frequencies vary among populations, and in certain cases, these differences may partly elucidate variations in disease prevalence. For instance, a North–South gradient in T1DM prevalence is generally observed across European countries, with Scandinavians at the highest risk. However, the Mediterranean island of Sardinia presents an exception, with a remarkably high T1DM risk. This elevated risk may be attributed in part to the increased frequencies of the T1DM-predisposing DR3 haplotype and the DR4 haplotype carrying DRB1*04:05, identified as the highest-risk DRB1*04 allele. Additionally, the frequency of the highly protective DR2 haplotype, DRB1*15:01-DQA1*01:02-DQB1*06:02, is lower in Sardinia compared to other Caucasian populations [4,5,6]. It has been reported as well that children with the highest-risk HLA genotype (DR3/DR4-DQB1*03:02) have approximately a 1 in 20 chance of being diagnosed with T1DM by age 15. Even more, if the child possesses this high-risk genotype and has a sibling with T1DM, the risk increases dramatically to around 55% [7,8,9]. Notably, these aspects were scarcely investigated in the Romanian T1DM population.

The role of HLA haplotypes, as the primary genetic determinant of T1DM, requires further investigation to better evaluate the risk and mitigate the progression of certain autoimmune diseases associated with this disease, as well documented in the literature, especially in presymptomatic individuals. The potential coexistence of autoimmune disorders within the same individual or familial context may be elucidated by a shared genetic foundation and compromised immune regulation. Routine screening for autoimmune endocrine pathologies, particularly for the descendants of individuals affected by T1DM and autoimmune thyroid diseases (AITD), is strongly advised [10,11].

This recommendation arises from research indicating significant etiological overlap between T1DM and Hashimoto’s thyroiditis. In an earlier study, for instance, genetic influences exhibited correlations between the two conditions, with 11% of the variance in T1DM and 9% in Hashimoto’s thyroiditis being explained by additive genetic effects common to both disorders. A recent Brazilian multicenter study, conducted on 1760 patients, found that the prevalence of autoimmune diseases among T1DM patients was 19.5%, while the prevalence of autoimmune thyroid diseases was 16.1% [12]. Furthermore, environmental factors unique to individual twins, yet shared across both diseases, contributed to 10% of the variance in type 1 diabetes and 18% in Hashimoto’s thyroiditis [13].

Celiac disease (CD) is another autoimmune disorder that shares a significant genetic predisposition with T1DM. The combined prevalence of CD in individuals with T1DM was determined to be 6.0% (95% CI = 5.0–6.9%). Age explained the heterogeneity, with a lower CD prevalence observed in adults compared to age-mixed samples encompassing both children and adults and in children alone [14]. A recent research study from South Africa revealed that 63.6% of their T1DM patients had a minimum of one high-risk HLA allele associated with CD, DQ2 being the most frequent [15].

The heightened prevalence of CD in children with T1DM suggests a connection beyond mere association. Evidence from both animal models and human studies supports the existence of a link between the gut immune system and T1DM [16].

In recent years, several studies have indicated a correlation between low levels of vitamin D and the increased incidence of T1DM. Oral supplementation of vitamin D emerges as a highly promising intervention for the prevention of this disease, given that 1,25OH vitamin D regulates over 200 genes associated with autoimmunity. Normalizing vitamin D levels has been observed to plateau the incidence of type 1 diabetes after years of escalation [17,18,19,20]. Children who were given vitamin D supplements were associated with a lower frequency of the T1DM rate ratio (RR) for regular versus no supplementation, and irregular versus no supplementation [20,21]. Interestingly, an inverse latitudinal gradient of global ultraviolet B (UVB) irradiance distribution has been reported, contributing to the causes of T1DM. An intriguing study highlights an inverse relationship between global ultraviolet B (UVB) irradiance and T1DM incidence rates. It suggests that regions with lower UVB levels due to geographic conditions tend to have higher rates of T1DM [10]. 

Collectively, these data suggest a potential association between specific HLA alleles characteristic of T1DM and vitamin D deficiency.

This study aims to extensively examine the HLA alleles and haplotypes in a cohort of pediatric patients with T1DM in Romania, to identify genetic predispositions to autoimmune diseases such as autoimmune thyroid disease and celiac disease, and to investigate the correlations of these genetic factors with vitamin D deficiency. To achieve this, children diagnosed with T1DM from the southern region of Romania underwent genotyping for the HLA A, B, C, DR, and DQ loci. Regression analysis was then employed to determine any correlation between the most prevalent predisposing and protective DR-DQ haplotypes and the onset of T1DM, autoimmunity, celiac disease, and low vitamin D levels.

## 2. Materials and Methods

### 2.1. Ethical Approval

This research was carried out at the Pediatric Endocrinology and Diabetes Department of Elias Emergency University Hospital in Bucharest, Romania, from 2019 to 2021. Prior approval for this study was obtained from the Ethics Commission for Scientific Research of the hospital under protocol code 1695 on 12 March 2019. All planning, data collection, and reporting involving human subjects were conducted in compliance with National and European regulations, including the Helsinki Declaration of 2013. Parental approval was obtained through written informed consent for the use of medical records in scientific research, following General Data Protection Regulation (GDPR) requirements.

### 2.2. Subjects and Study Design

This was an analytical retrospective cross-sectional study of 36 T1DM pediatric patients 1–18 years of age at onset, of Caucasian origin (defined by Steenkiste A. et al.) [2]. The inclusion criteria were as follows: (1) a positive T1DM diagnosis, according to the American Diabetes Association criteria [15], and (2) clinical expression of the disease during the six months preceding admission. Exclusion criteria comprised (1) patients with other types of diabetes mellitus (neonatal, type 2, MODY), (2) T1DM patients with a known diagnosis for more than 6 months, and (3) antibiotic administration in the last month before the admission to the department.

Patients were either directly attended to by their primary care physicians or were discharged or transferred to the Pediatric Endocrinology and Diabetes Department at the Elias Emergency University Hospital in Bucharest from other pediatric hospitals in the southern region of Romania. This facility is among the three designated reference centers for the assessment of continuous glucose monitoring devices and insulin pumps, which are covered by reimbursement from the National Health System in the southern region. Taking this into consideration, a majority of recently diagnosed T1DM patients seek assistance from this institution. The patients were recruited in chronological order of hospitalization, provided they met the inclusion criteria and their parents consented to participate in this study.

The date of T1DM diagnosis with corresponding glycated A1c hemoglobin (HbA1c) and blood glucose values were assessed, as well as blood glucose control, and details regarding the type of treatment received, including the presence of sensors and pumps.

Data were systematically gathered for each individual diagnosed with T1DM through a comprehensive patient form.

### 2.3. Laboratory Procedures

Two 10 cc peripheral blood samples were obtained eight hours after the last meal for blood chemistry and HLA genotyping.

Blood glucose and HbA1c were assessed. Thyroid function was evaluated through thyroid-stimulating hormone (TSH) and free thyroxine (free T4) measurements and the thyroid autoimmunity by anti-thyroglobulin and anti-thyroid peroxidase antibodies. The anti-tissue transglutaminase antibodies for celiac disease were dosed. Additionally, 25(OH) vitamin D, and calcium-phosphorus metabolism were evaluated, with a determination of total serum calcium, phosphorus, and parathyroid hormone (PTH).

Various analytical methods were employed for these assessments. Specifically, ELISA methods were used for 25(OH) vitamin D, anti-thyroglobulin, anti-thyroid peroxidase, and anti-tissue transglutaminase antibody measurements [22]. Spectrophotometric methods were utilized for lipid, glucose, creatinine, aspartate aminotransferase (AST), alanine aminotransferase (ALT), calcium, and phosphorus measurements on a Jasco V 650 spectrometer, with all reagents supplied by Sigma-Aldrich, St. Louis, MO, USA [23].

TSH and free thyroxine (fT4) levels were evaluated through electrochemiluminescence immunoassay (ECLIA) methods on a Roche COBAS e 411 analyzer, supplyed by Roche, Switzerland, using kits from Immunodiagnostic Systems, East Boldon, UK. HbA1c was determined using the high-performance liquid chromatography (HPLC) method, certified by the National Glycohemoglobin Standardization Program (NGSP), with the BIO-DAD d10, supplied by Jensen Pharma, Bucharest, Romania. The blood glucose was measured using spectrophotometric assays on a Jasco V 650 spectrometer. All reagents were supplied by Sigma-Aldrich, St. Louis, MO, USA [24,25].

### 2.4. HLA Genotyping

Peripheral blood samples were collected from participants, DNA extraction was performed from fresh whole blood using magnetic beads-based reagents, innuPREP Blood DNA Mini Kit-IPC16 (Analytik Jena GmbH, Jena, Germany), compatible with an automated instrument InnuPure C16 (Analytik Jena GmbH, Jena, Germany). HLA typing was done by a hybrid capture-based NGS method and AlloSeq Tx17 reagents were used for library preparation, according to the manufacturer’s protocol (CareDx, Inc., Brisbane, CA, USA), followed by sequencing on the Illumina MiniSeq (Illumina Inc., San Diego, CA, USA) platform. The targeted loci were HLA-A, B, C, DRB1, DQB1, DQA1, and DPB1. The allelic resolution was achieved through sequence-based typing (Sanger) methods, and haplotypes were reconstructed using the Assign ver. 1.0.4 software, IMGT/HLA 3.49. Individual extended genotypes were derived from reconstructed haplotypes. Patients were classified based on their haplotypes, with controls comprising non-diabetic individuals from Romanian patients and relatives screened for bone marrow donation [26,27].

### 2.5. Statistical Analysis

A binary logistic regression analysis evaluating the association between HLA alleles and haplotypes, anti-thyroglobulin antibodies, anti-thyroid peroxidase (ATPO) antibodies, anti-transglutaminase antibodies, and vitamin D deficit was performed using SPSS Windows v.17.0 (SPSS v 29.0, Inc., Chicago, IL, USA). A power analysis test was used, to estimate the minimum sample size needed and to exclude that the results are due to chance, with a result of 0.82 for our sample size. A Bonferroni correction was used for multiple genetic testing, adjusting the *p* values correspondingly, to reduce the risk of type 1 error appearance. This statistical test was used because multiple hypotheses were carried out, regarding the associations between HLA alleles and haplotypes and other patient variables. For all tests, *p* < 0.05 was considered statistically significant.

## 3. Results

### 3.1. Clinical and Biological Characteristics of the Patients

The participants had a mean age at onset of 8.72 ± 0.759 years old (median: 8 years old), with a male-to-female ratio of 1.11. The mean weight was 39.34 ± 3.59 kg and the median was 33.35 kg. Their mean height was 138.15 ± 5.94 cm with a median of 141.3 cm (+2 SD). The average body mass index (BMI) was 17.62 ± 0.59 kg/m^2^ (−1.5 SD for Romanian children) and the median was 16.7 kg/m^2^ [28]. At the onset, the mean HbA1c level was 11.57 ± 0.43% (median: 11.4%) and the average glycemia was 335.52 ± 23.87 mg/dL (median: 323 mg/dL). The gender ratio was balanced, not skewed towards males as reported in the literature, with a prepubertal onset age. Although glycemia and HbA1c at diagnosis were elevated, they did not adversely impact the linear growth of the children [29].

Eleven individuals had a first-degree relative with T1DM, while fifteen had a first-degree relative with autoimmune disorders. Three patients were diagnosed with Hashimoto’s thyroiditis, and ATPO antibodies were found in ten patients (27.7%), while ATG antibodies were detected in seven patients, six of whom had both types. Low vitamin D levels were observed in 23 patients, and only 2 patients tested positive for anti-transglutaminase antibodies.

Regarding the treatment, 15 patients used a continuous glucose monitoring system, with only 4 using an insulin pump. The mean insulin dose per kg of body weight over 24 h was 26.1 ± 4.4 UI, with a median of 2.41 UI.

The complete biochemical evaluation is presented in Table 1.

### 3.2. Protecting HLA Allele and Haplotypes

**Figure 1:**
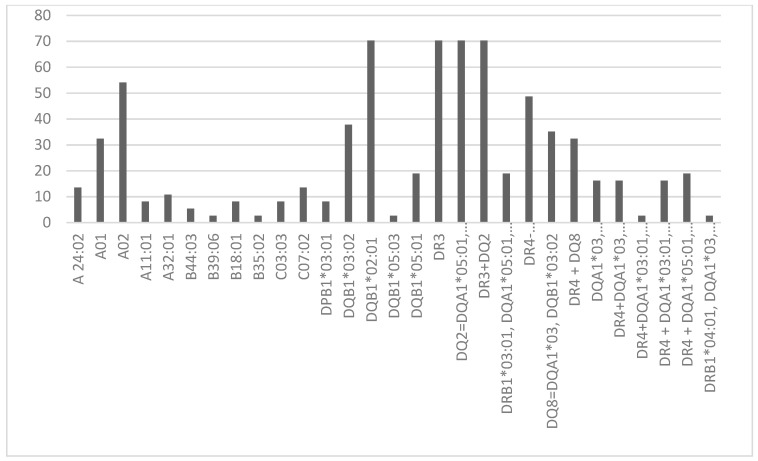
Frequency of predisposing HLA alleles and haplotypes.

**Figure 2:**
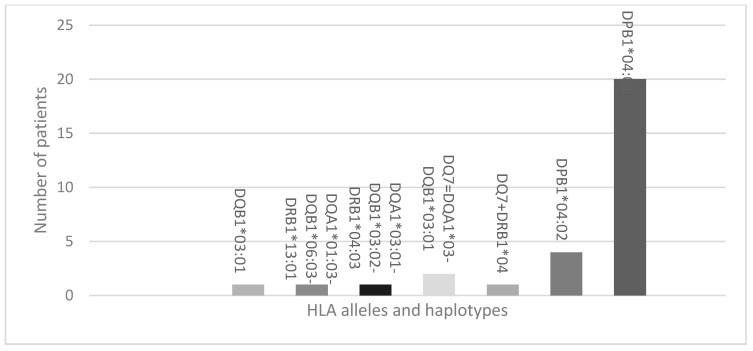
Frequency of protecting HLA alleles and haplotypes.

The most frequent predisposing alleles and haplotypes identified were as follows: DR3 (70.27%), DQ2 (70.27%), DR3 + DQ2 (70.27%), DQB1*02:01 (70.27%), A02 (54.05%), DR4 (48.65%), DQB1*03:02 (37.84%), DQ8 (35.14%), A01 (32.43%), DR4 + DQ8 (32.43%). Additionally, the most prevalent protecting allele was DPB1*04:01 (52.63%). These alleles and haplotype frequencies are visualized in Figure 1 and Figure 2.

### 3.3. HLA Alleles and Haplotypes Linked to Elevated ATPO and ATG Levels

Positive correlations between the absence of protective HLA alleles and the presence of ATPO antibodies were found. Specifically, the absence of DPB1*04:02 (score = 4.4, *p* = 0.036) and DPB1*04:01 (score = 9.491, *p* = 0.002) showed such correlations, as illustrated in Table 2. Following adjustments with the Bonferroni correction, the significance level was recalibrated to 0.027.

Furthermore, we identified positive correlations between the absence of DPB1*04:01 and the presence of anti-thyroglobulin antibodies (score = 4.702, *p* = 0.03), as detailed in Table 3. After applying the Bonferroni correction, the significance level was adjusted to 0.026.

### 3.4. HLA Alleles and Haplotypes Correlated with Positive Transglutaminase Antibodies

We observed a positive correlation between positive transglutaminase antibodies and predisposing alleles and haplotypes, as shown in Table 4. Following adjustment with the Bonferroni correction, the significance level was revised to 0.023.

### 3.5. HLA Alleles and Haplotypes Associated with Vitamin D Deficiency

The HLA allele DPB1*03:01 was associated with a deficiency in 25(OH) vitamin D levels (*p* = 0.021), as indicated in Table 5. After applying the Bonferroni correction, the significance level was adjusted to 0.021.

## 4. Discussions

### 4.1. Protecting and Predisposing Alleles and Haplotypes in T1DM Patients

Our investigation into the HLA alleles and haplotypes among Romanian T1DM patients revealed distinct patterns of genetic susceptibility to autoimmune thyroid disease, celiac disease, and vitamin D deficiency. Notably, the high prevalence of DR3, DQ2, and DR3 + DQ2 haplotypes, found in 70.27% of our cohort, contrasts with the broader DR3/DR4 haplotype distribution reported in Scandinavian populations (12.5–34%) [6,29,30]. Interestingly, while the protective haplotype DRB1*15:01-DQA1*01:02-DQB1*06:02 common in other regions was absent, the protective allele DPB1*04:01 was detected in over half of our patients (52.63%) [30]. These findings underscore unique regional variations in HLA-associated T1DM susceptibility, emphasizing the need for geographically tailored genetic studies.

There are only two studies conducted on the Romanian population that exhibit HLA susceptibility to T1DM, made in the early 2000s. One of them concluded that the HLA allele DQB1 is predisposing for T1DM [31,32]. The other study underlined the strong connection between the presence of DQB1*02-DRB1*03, DQB1*03:02, the lack of DQB1*06:02 HLA class II alleles, and the diagnosis of T1DM in Romanian families [33]. The necessity and importance of the actual study lies in the fact that there has not been any other research conducted and published since then in Romania regarding an HLA connection to T1DM.

### 4.2. Thyroid Autoimmunity and T1DM

The link between T1DM and thyroid autoimmunity was further supported by the prevalence of Hashimoto thyroiditis in 27.7% of our patients, aligning with previous Romanian pediatric studies [34,35]. Our data suggest a genetic connection between T1DM and thyroid autoimmunity, highlighted by the association of certain HLA haplotypes with the presence of anti-thyroid peroxidase (ATPO) and anti-thyroglobulin (ATG) antibodies, with the absence of DPB1*04:02 and DPB1*04:01, and the correlation of positive anti-thyroglobulin (ATG) antibodies with the absence of DPB1*04:01, found in the current study population. Additionally, DRB1*04:05-DQB1*04:01, a major haplotype of T1DM individuals, was more frequent in the T1DM patient cohort compared to the healthy cohort (OR 3.08, *p* < 0.0001). These haplotypes were not found to be statistically associated with AITD in our children cohort, even though they were frequently reported amongst the patients with T1DM [31,36,37].

This overlap of autoimmune profiles suggests shared genetic pathways, which could inform targeted screening and management strategies in T1DM patients predisposed to thyroid disorders

### 4.3. Vitamin D Deficiency and T1DM

Vitamin D deficiency was highly prevalent among our T1DM cohort (83%), corroborating global research indicating a widespread issue among T1DM patients [35,38,39]. The correlation between DPB1*03:01 and vitamin D levels below 30 ng/mL highlights potential genetic markers that could predict deficiency risks. Addressing vitamin D deficiency through dietary supplementation could thus be a promising preventative strategy for mitigating T1DM progression, supported by its regulatory role in over 200 genes linked to autoimmunity.

In our group, DPB1*03:01 correlates with vitamin D deficit (<30 ng/mL). Additionally, these findings support existing indications of a connection between low 25(OH) vitamin D levels and an increased incidence of T1DM, with DPB1*03:01 correlating with vitamin D deficiency. Oral intake may be one of the most promising candidates for the prevention of T1DM, as 25(OH) vitamin D regulates more than 200 genes and autoimmunity. By normalizing vitamin D levels, the incidence of T1DM plateaued after years of rising levels, as revealed in research conducted on northern populations [10,19,20,21].

Understanding the mechanisms through which vitamin D mitigates specific adverse health outcomes is essential for comprehending deficiencies and should be consistently taken into account when planning future trials. The knowledge that correcting deficiencies can alleviate tissue dysfunction in numerous prevalent conditions underscores the case for enhancing the availability of vitamin D in populations often impacted by a vitamin D deficit [11].

### 4.4. Celiac Disease and T1DM

The association between T1DM and celiac disease was evident through the identification of specific HLA alleles correlating with anti-transglutaminase antibodies. These genetic markers, distinct yet partially overlapping with those linked to thyroid autoimmunity, suggest a complex interplay between T1DM and celiac disease. This reinforces the need for comprehensive screening for celiac disease in T1DM patients, particularly given the significant overlap in genetic susceptibility [40].

The alleles positively correlated with positive anti-transglutaminase antibodies (C03:03, *p* = 0.026; DRB1*04:01-DQA1*03-DQB1*03:01, *p* = 0.0001) and the lack of DQA1*01:03-DQB1*06:03-DRB1*13:01 (*p* < 0.0001) are slightly different from those that correlate with autoimmune thyroiditis (the lack of DPB1*04:02, *p* = 0.036 and DPB1*04:01, *p* = 0.002). Also, the alleles correlated with positive anti-transglutaminase antibodies are common to those that correlate with positive ATG antibodies in autoimmune thyroiditis, namely the lack of DPB1*04:01 (*p* = 0.03). The same alleles, DRB1*03:01, DRB1*04, and DRB3*01:07/15, were present in patients with T1DM and CD in a recent study from North India, thus suggesting that these HLA genes are susceptible to both diseases [41].

The identification of specific HLA alleles positively correlating with anti-transglutaminase antibodies in these Romanian patients may shed light on the association between celiac disease and T1DM.

### 4.5. Future Research

Our findings pave the way for further studies to explore the modulation of autoreactivity within the immune system through detailed genetic and cellular analyses. Future research should also include a larger, more diverse cohort to validate these preliminary insights and expand on the connections between T1DM, autoimmune disorders, and nutrient deficiencies.

Overall, genetic analyses, combined with an in-depth understanding of cell signaling pathways involved in co-stimulation and regulatory networks, could unveil a novel insight: the potential modulation of autoreactivity levels within the immune system [42,43].

All these interconnected findings suggest a genetic autoimmune transmission. Unfortunately, there have not been any studies conducted on the Romanian pediatric T1DM population regarding the genetic association of HLA alleles and haplotypes with autoimmune disorders and vitamin D deficiency.

Acknowledgment of several limitations in the current research is necessary. This study included a relatively small number of subjects, which could potentially affect the strength of the correlations identified. The limited patient pool can be attributed to both parental reluctances to enroll their children in this study and to the COVID-19 2020–2021 pandemic restrictions which affected the hospital presentation of chronic patients during the period in which this study was performed. However, our research was originally designed as a pilot cross-sectional study, and the obtained data provide a strong foundation for further research, indicating the necessity for larger-scale investigations to validate these initial insights. Furthermore, additional limitations arise from the retrospective nature of this study and the heterogeneity of the participant group. The heterogeneity among patients, ranging from 1 to 18 years old, without any further selection criteria beyond recent onset, and division into age groups, provided a comprehensive overview of the individuals enrolled in this study, underscoring the importance of exploring the genetic connection between T1DM and autoimmune diseases. This connection was revealed despite the small mixed group of patients. As this research does not include a healthy control group as per its original study design, it serves as a pilot study that sheds light on the relationship between HLA haplotypes among T1DM patients and autoimmune disorders. Looking ahead, we anticipate future research that includes a healthy control group to gather more accurate data for comparison.

## 5. Conclusions

Our findings demonstrate a correlation between predisposing T1DM alleles and haplotypes and the presence of anti-transglutaminase antibodies and anti-thyroid antibodies, indicating a genetic predisposition to autoimmune transmission. Also, the connection between T1DM and vitamin D deficit should raise awareness regarding screening procedures and efficient treatment. As we found that the absence of some protective genes for T1DM, associated with clinical onset, also increases the risk for other autoimmune diseases related to the HLA DR3, we propose that immunological screening for these conditions be conducted more frequently among these patients. Consequently, genetic analysis could be the key to predict the onset of latent autoimmune diseases, in order to prevent their development amongst T1DM patients.

## Figures and Tables

**Table 1:** Biochemical parameters of the patients.

	Mean and Std. Error	Median	Normal Values
AST (U/L)	23.11 ± 1.12	22	13–26
ALT (U/L)	19.5 ± 1.56	16.5	8
Creatinine (mg/dL)	0.68 ± 0.02	0.64	0.57–0.86
Total-cholesterol (mg/dL)	173.5 ± 8.19	164.5	140–200
LDL-cholesterol (mg/dL)	104.22 ± 6.11	95.2	<130
HDL-cholesterol(mg/dL)	61.74 ± 2.05	61	>40
Triglycerides (mg/dL)	54.06 ± 3.38	50.5	35–150
TSH (μUI/mL)	2.54 ± 0.2	2.41	0.3–3.6
fT4 (ng/dL)	1.18 ± 0.02	1.2	0.8–1.48
25(OH) vitamin D (ng/mL)	29.47 ± 2.12	24.3	>30
Total calcium (mg/dL)	9.48 ± 0.07	9.55	8.4–10.2
Phosphorus (mg/dL)	4.74 ± 0.12	4.8	2.5–4.5
PTH (pg/mL)	33.37 ± 2.96	28.18	15–65
ATPO (IU/mL)	58.85 ± 29.08	9	<16
ATG (IU/mL)	63.6 ± 26.64	10.19	<20
Anti-transglutaminase antibodies (IU/mL)	0.882 ± 0326	0.45	<10

**Table 2:** HLA alleles and haplotypes associated with positive ATPO values (>16 UI/mL).

Alleles and Haplotypes	Score	*p*-Value
A 24:02	2.536	0.111
A01	0.190	0.663
A02	0.579	0.447
A11:01	1.381	0.240
A32:01	0.048	0.827
B44:03	0.416	0.519
B39:06	0.435	0.510
B18:01	1.897	0.168
B35:02	0.435	0.510
C03:03	2.132	0.144
C07:02	0.292	0.589
DPB1*03:01	0.020	0.887
DQB1*03:02	0.010	0.919
DQB1*02:01	1.211	0.271
DQB1*05:03	0.435	0.510
DQB1*05:01	0.006	0.941
DR3	1.211	0.271
DQ2 = DQA1*05:01-DQB1*02:01	1.211	0.271
DR3 + DQ2	1.211	0.271
DRB1*03:01-DQA1*05:01-DQB1*03:02	0.585	0.444
DR4-04:01/04:02/04:04/04:05/04:08	1.973	0.160
DQ8 = DQA1*03-DQB1*03:02	0.190	0.663
DR4 + DQ8	0.045	0.832
DQA1*03-DQB1*03:04/DQB1*02	1.408	0.235
DR4 + DQA1*03-DQB1*03:04/DQB1*02	1.408	0.235
DR4 + DQA1*03:01-DQB1*04:01	2.429	0.119
DR4 + DQA1*03:01-DQB1*02:01	0.262	0.609
DRB1*04:01-DQA1*-DQB1*03:01	2.429	0.119
Absence of DQA1*03:01-DQB1*03:02-DRB1*04:03	0.435	0.510
Absence of DQA1*01:03-DQB1*06:03-DRB1*13:01	2.429	0.119
Absence of DQ7 = DQA1*03-DQB1*03:01	0.416	0.519
Absence of DQ7 + DRB1*04	0.435	0.510
Absence of DPB1*04:02	4.400	0.036
Absence of DPB1*04:01	9.491	0.002

**Table 3:** HLA alleles and haplotypes associated with ATG values (>20 IU/mL).

Alleles and Haplotypes	Score	*p*-Value
A 24:02	1.062	0.303
A01	0.08	0.777
A02	0.39	0.532
A11:01	0.935	0.334
A32:01	0.02	0.887
B44:03	0.945	0.331
B39:06	0.294	0.588
B18:01	1.286	0.257
B35:02	0.294	0.588
C03:03	3.74	0.053
C07:02	0.017	0.898
DPB1*03:01	0.234	0.629
DQB1*03*02	0.009	0.926
DQB1*02*01	1.286	0.257
DQB1*05*03	0.294	0.588
DQB1*05*01	0.129	0.72
DR3	1.286	0.257
DQ2 = DQA1*05*01-DQB1*02*01	1.286	0.257
DR3 + DQ2	1.286	0.257
DRB1*03:01-DQA1*05:01-DQB1*03:02	0.129	0.72
DR4-04:01/04:02/04:04/04:05/04:08	0.963	0.326
DQ8 = DQA1*03-DQB1*03:02	0.321	0.571
DR4 + DQ8	0.15	0.699
DQA1*03-DQB1*03:04/DQB1*02	0.514	0.473
DR4 + DQA1*03-DQB1*03:04/DQB1*02	0.514	0.473
DR4 + DQA1*03:01-DQB1*04:01	0.294	0.588
DR4 + DQA1*03:01-DQB1*02:01	0.017	0.898
DR4 + DQA1*05:01-DQB1*03:02	0.129	0.72
DRB1*04:01-DQA1*03-DQB1*03:01	3.6	0.058
Absence of DQA1*01:03-DQB1*06:03-DRB1*13:01	3.6	0.058
Absence of DQ7 = DQA1*03-DQB1*03:01	0.945	0.331
Absence of DQ7 + DRB1*04	0.294	0.588
Absence of DPB1*04:02	0.02	0.887
Absence of DPB1*04:01	4.702	0.03

**Table 4:** Alleles and haplotypes associated with positive anti-transglutaminase antibodies (>10 IU/mL).

Alleles and Haplotypes	Score	*p*-Value
A 24:02	2.408	0.121
A01	1.015	0.314
A02	1.797	0.18
A11:01	0.187	0.666
A32:01	0.256	0.613
B44:03	0.121	0.728
B39:06	0.059	0.809
B18:01	0.256	0.613
B35:02	0.059	0.809
C03:03	4.98	0.026
C07:02	2.408	0.121
DPB1*03:01	0.187	0.666
DQB1*03:02	1.145	0.285
DQB1*02:01	0.298	0.585
DQB1*05:03	0.059	0.809
DQB1*05:01	0.493	0.482
DR3	0.298	0.585
DQ2 = DQA1*05:01-DQB1*02:01	0.298	0.585
DR3 + DQ2	0.298	0.585
DRB1*03:01-DQA1*05:01-DQB1*03:02	0.409	0.522
DR4-04:01/04:02/04:04/04:05/04:08	0.014	0.906
DQ8 = DQA1*03-DQB1*03:02	1.015	0.314
DR4 + DQ8	0.895	0.344
DQA1*03-DQB1*03:04/DQB1*02	0.409	0.522
DR4 + DQA1*03-DQB1*03:04/DQB1*02	0.409	0.522
DR4 + DQA1*03:01-DQB1*04:01	0.059	0.809
DR4 + DQA1*03:01-DQB1*02:01	0.33	0.565
DR4 + DQA1*05:01-DQB1*03:02	0.409	0.522
DRB1*04:01-DQA1*03-DQB1*03:01	17.986	<0.001
Absence of DQA1*0301-DQB1*03:02-DRB1*04:03	0.059	0.809
Absence of DQA1*0103-DQB1*06:03-DRB1*13:01	17.986	<0.01
Absence of DQ7 = DQA1*03-DQB1*03:01	0.121	0.728
Absence of DQ7 + DRB1*04	0.059	0.809
Absence of DPB1*04:02	0.256	0.613

**Table 5:** HLA alleles and haplotypes associated with vitamin D deficit (<30 ng/mL).

Alleles and Haplotypes	Score	*p*-Value
A 24:02	1.207	0.272
A01	1.117	0.291
A02	1.137	0.286
A11:01	1.987	.0159
A32:01	0.282	0.595
B44:03	3.473	0.062
B39:06	0.626	0.429
B18:01	0.282	0.595
B35:02	0.626	0.429
C03:03	0.028	0.867
C07:02	1.207	0.272
DPB1*03:01	5.363	0.021
DQB1*03:02	0.426	0.514
DQB1*02:01	1.244	0.265
DQB1*05:03	0.626	0.429
DQB1*05:01	0.092	0.761
DR3	1.244	0.265
DQ2 = DQA1*05:01-DQB1*02:01	1.244	0.265
DR3 + DQ2	1.244	0.265
DRB1*03:01-DQA1*05:01-DQB1*03:02	0.450	0.502
DR4-04:01/04:02/04:04/04:05/04:08	0.087	0.769
DQ8 = DQA1*03-DQB1*03:02	0.153	0.695
DR4 + DQ8	0.014	0.904
DQA1*03-DQB1*03:04/DQB1*02	1.365	0.243
DR4 + DQA1*03-DQB1*03:04/DQB1*02	1.365	0.243
DR4 + DQA1*03:01-DQB1*04:01	0.626	0.429
DR4 + DQA1*03:01-DQB1*02:01	0.011	0.915
DR4 + DQA1*05:01-DQB1*03:02	0.450	0.502
DRB1*04:01- DQA1*03-DQB1*03:01	1.688	0.194
Lack of DQA1*03:01-DQB1*03:02- DRB1*04:03	0.626	0.429
Lack of DQA1*01:03-DQB1*06:03- DRB1*13:01	1.688	0.194
Absence of DQ7 = DQA1*03- DQB1*03:01	0.133	0.715
Absence of DQ7 + DRB1*04	0.626	0.429
Absence of DPB1*04:02	2.633	0.105

## Data Availability

All data are available upon request.
